# The Traditional Chinese Medicine Formula Jing Guan Fang for Preventing SARS-CoV-2 Infection: From Clinical Observation to Basic Research

**DOI:** 10.3389/fphar.2022.744439

**Published:** 2022-03-21

**Authors:** Yueh-Hsin Ping, Hsin Yeh, Li-Wei Chu, Zhi-Hu Lin, Yin-Chieh Hsu, Lie-Chwen Lin, Chung-Hua Hsu, Shu-Ling Fu, Tung-Yi Lin

**Affiliations:** ^1^ Department and Institute of Pharmacology, National Yang Ming Chiao Tung University, Taipei, Taiwan; ^2^ Institute of Biophotonics, National Yang Ming Chiao Tung University, Taipei, Taiwan; ^3^ Institute of Traditional Medicine, National Yang Ming Chiao Tung University, Taipei, Taiwan; ^4^ National Research Institute of Chinese Medicine, Taipei, Taiwan; ^5^ Branch of Linsen Chinese and Kunming, Taipei City Hospital, Taipei, Taiwan; ^6^ Biomedical Industry Ph.D. Program, National Yang Ming Chiao Tung University, Taipei, Taiwan

**Keywords:** Chinese medicine decoction, Jing Guan Fang, ACE2, TMPRSS2, COVID-19, prevention

## Abstract

COVID-19 is a global epidemic. Developing adjuvant therapies which could prevent the virus from binding to cells may impair viral infection. This study produces a traditional Chinese medicine formula, Jing Guan Fang (JGF), based on ancient medical texts, and examines the efficacy and the mechanism by which JGF prevents viral infections. JGF reduces COVID-19 like symptoms. Functional studies show that JGF inhibits the formation of syncytium and reduces the formation of viral plaque. JGF is not toxic *in vitro* and *in vivo*. Mechanistically, JGF induces lysosomal-dependent ACE2 degradation and suppresses mRNA and the protein levels of TMPRSS2 in human lung WI-38 and MRC-5 cells. Mice that inhale JGF exhibit reduced ACE2 and TMPRSS2 protein levels in lung tissues. Together, these findings suggest that JGF may improve the COVID-19 like symptoms and inhibit viral infection. Moreover, JGF may be applicable as an adjuvant preventive strategy against SARS-CoV-2 infection in addition to the use of vaccines.

## Introduction

Since December 2019, the severe acute respiratory syndrome coronavirus 2 (SARS-CoV-2) infection has caused coronavirus disease 2019 (COVID-19), which is a severe pandemic with a wide spectrum of clinical symptoms, ranging from asymptomatic infection or a mild, flu-like illness to life-threatening diseases including severe pneumonia and severe acute respiratory syndrome (SARS) (Collaborators, 2020; Guan et al., 2020; Zhu et al., 2020). Until mid-May 2021, more than 162 million COVID-19 cases were recorded worldwide. More than 3.3 million cases resulted in death tolls (WHO, 2021). SARS-CoV-2, which is the largest positive-strand RNA virus, is a member of the Coronaviridae family which includes the severe acute respiratory syndrome coronavirus (SARS-CoV) and Middle East respiratory syndrome coronavirus (MERS-CoV) (Collaborators, 2020; Zhou et al., 2020). The RNA genome of the SARS-CoV-2 is approximately 30 K nucleotides long and encodes four structure proteins, including a spike glycoprotein (S), an envelope protein (E), a membrane protein (M), and a nucleoprotein (N) (Chen et al., 2020). The S protein is composed of two functional subunits: S1 and S2. The S1 subunit has a receptor binding domain (RBD) at the N-terminal of the S protein, which binds to a critical host receptor: the angiotensin-converting enzyme-2 (ACE2). The S2 subunit has a fusion domain at the C-terminal of the S protein that fuses viral and cellular membranes (Li, 2016; Yan et al., 2020). The SARS-CoV-2 infection begins when virus particles attach to host cells due to interactions between S1 and ACE2 (Lan et al., 2020; Walls et al., 2020; Wang et al., 2020; Wrapp et al., 2020). The S2 subunit is further cleaved by host proteases, including the transmembrane serine protease 2 (TMPRSS2) and furin, which results in the dissociation of the S1 and the S2-mediated membrane fusion process (Cheng et al., 2020; Shang et al., 2020; Li et al., 2021). The S protein must bind with ACE2 and the S protein must be proteolyzed by membrane proteases if the SARS CoV-2 and host cell membrane are to be fused so an ability to block connections between the virus and host cells is a necessary facet of anti-SARS-CoV-2 agents.

Vaccines have been developed. Development of additional strategies/adjuvant therapies to prevent frontline medical staff from contacting COVID-19 may be an important priority. Traditional Chinese medicine (TCM) is arguably the world’s oldest, continually practiced medical modality. Chinese herbal medicine is an integral part of TCM dealing with natural products, that when used in combination become multi-functional. Western medicine explores a single compound and has a clear structure and anti-disease mechanism but Chinese herbal medicine is a comprehensive collection of various compounds and various functions. Some studies show that herbal medicine can reduce viral infection (Yang et al., 2020; Jan et al., 2021; Tsai et al., 2021). Chinese herbal medicine uses a cocktail-like effect to block viral infections due to their complex contents. Using clinical symptomatology, the authors’ collective experience during the 2003 SARS outbreak and with reference to the ancient medical text, *WenYi Lun* (溫疫論), this study designs a TCM formula, Jing Guan Fang (JGF), to prevent viral infection. JGF consists of five commonly used herbs: *Forsythia suspensa* (Thunb.) Vahl (as the Sovereign drug; 君藥), *Scutellaria baicalensis* Georgi (as the Minister drug; 臣藥), *Bupleurum chinese* DC (as the Assistant drug; 佐藥), *Magnolia officinalis* Rehder and E.H. Wilson (as the Assistant drug; 佐藥) and *Agastache rugose* (Fisch. and C.A. Mey.) Kuntze (as the Courier drug; 使藥).

This study determines the beneficial effects of JGF in improving clinical Covid-19 like symptoms and studies how JGF affects SARS-CoV-2 spike protein/ACE2 interaction and reduces plaque formation of SARS-CoV-2. The mechanism by which JGF downregulates ACE2 and TMPRSS2 levels in lung cells *in vitro* and in lung tissues of a mouse model *in vivo* is also determined. A high-performance liquid chromatography (HPLC) fingerprint of JGF was performed to ensure authentication and standardization.

## Materials and Methods

### Jing Guan Fang Preparation

JGF was formulated by our herbal medicine physicians using herbal medicine theory and clinical experience (Cheng et al., 2006; Deng et al., 2012; Law et al., 2017; Ghildiyal et al., 2020; Yang et al., 2020). The formula for JGF uses five herbs: 10 g of *Forsythia suspensa* (Thunb.) Vahl [Oleaceae] (30.3% of total weight), 8 g of *Scutellaria baicalensis* Georgi [Labiatae] (24.2% of total weight), 6 g of *Bupleurum chinense* DC. [Umbelliferae] (18.2% of total weight), 6 g of *Magnolia officinalis* Rehder & E.H.Wilson var. *biloba* Rehder & E.H.Wilson [Magnoliaceae] (18.2% of total weight) and 3 g of *Agastache rugosa* (Fisch. & C.A.Mey.) Kuntze [Labiatae] (9.0% of total weight). All ingredients were purchased from a certificated pharmaceutical company (KO DA Pharmaceutical Co., Ltd. Taiwan). JGF is produced by the Branch of Linsen Chinese and Kunming, Taipei City Hospital (Taipei, Taiwan). All herbs were soaked in water and subsequently boiled for 4 h using an automatic herb boiling machine. The final product is 110 g/pack in weight and 100 ml/pack in volume. HPLC was performed to ensure the quality and standard contents of JGF (Supplementary Figure S1). To ensure the quality of JGF, the herbal-medicine pharmacists who participated in the study underwent training in the preparation of JGF. All JGF users were instructed how to take JGF. Non-symptomatic individuals were advised to take one dose a week and those who displayed Covid-19 like symptoms were advised to take two doses a week.

### Clinical Setting and Participants

JGF was initially designed for front line staff as a complementary preventative measure against Covid-19. The formula for JGF was then made freely available to the public on 20 February 2020, in five public hospitals in the Taipei area. The clinical study was conducted from 20 February 2020 to 20 May 2020. JGF was taken by subjects as a complementary preventative strategy. A total of 2468 packs of JGF were provided to 1086 individuals, of which 396 individuals participated in the questionnaire. All participants were from the Taipei area (Taipei city and New Taipei city). The protocol was approved by the Taipei City Hospital Institutional Review Board (TCHIRB-10904015) with Clinical Trial gov. Trial registration: NCT04388644, Registered 06 April 2020 - Retrospectively registered, https://clinicaltrials.gov/ct2/show/NCT04388644.

### Survey and Data Collection

Subjects filled out the online questionnaire voluntarily, or in person at the hospitals. The questionnaire recorded an individual’s demographic information, symptoms prior to taking JFG, and any improvement in symptoms or adverse effects and satisfaction.

### Cell Culture and Virus

BHK-21 cells and Calu-3 cells were cultured in Dulbecco’s Modified Eagle Medium (DMEM, Gibco) supplemented with 10% Fetal Bovine Serum (FBS) and 1 × penicillin/streptomycin solution. Vero E6 cells were maintained in High glucose DMEM (GeneDireX) supplemented with 10% FBS. Normal human lung WI-38 VA-13 subline 2RA and MRC-5 cells were purchased from the Bioresource Collection and Research Center (BCRC, Hsinchu, Taiwan). Cells were cultured in Minimum essential medium (Eagle, Gibco) supplemented with 10% FBS, 2 mM L-glutamine, 1.5 g/L sodium bicarbonate, 0.1 mM non-essential amino acids, and 1.0 mM sodium pyruvate at 37°C under a mixture of 95% air and 5% CO_2_. The SARS-CoV-2 strain 3586 (TSGH_15 GISAID accession number EPI_ISL_436100) was isolated at the Institute of Preventive Medicine, National Defense Medical Center and amplified in Vero E6 cells. The viral titer was determined using a plaque assay. SARS-CoV-2 was handled in a BSL-3 laboratory.

### The Cell-Cell Fusion Assay

Calu-3, as target cells, were seeded in a 12-well plate (1 × 10^6^ per well) and formed a single-layer of cell films for 48 h. BHK-21 cells were seeded in a 6-well plate (4 × 10^5^ per well) and transfected with both enhanced green fluorescent protein (GFP) and Spike plasmids at a ratio of 1:5 for 24 h. GFP/Spike-coexpressing BHK cells were harvested using Cell Dissociation Buffer (Gibco) and resuspended in serum free DMEM. For Spike-mediated cell–cell fusion assays, GFP/Spike-coexpressing BHK-21 cells, as donor cells, were added to Calu-3 cells, and incubated at 4°C for 45 min to allow cell-cell binding. They were then washed with PBS and the growth medium was replaced and the cells were then incubated at 37°C for 4 h to allow cell-cell fusion. After incubation, five fields were randomly selected in each well to record the GFP-expressing cell images using an inverted fluorescence microscope (Olympus IX70). The extension area of the GFP-expressing cell images was quantified to determine the degree of cell-cell fusion using ImageJ software. The fold change in the GFP area in control terms from 0 to 4 h was delimited to 100% fusion efficiency, and the fold changes in the GFP area for various JGF treatments were normalized to the control.

$$The\;normalized\;percantage\;\left(\%\right)=\frac{the\;fold\;change\;of\;GFP\;area\;}{the\;fold\;change\;of\;GFP\;area\;in\;control}\times 100$$

### SARS-CoV-2 Plaque Formation Assay

Vero E6 cells (4 × 10^5^/well) were seeded into 12-well plates. Before infection with SARS-CoV-2, train 3586, cells were treated with JGF (50, 100 and 200 μg/ml) for 3 h at 37°C and 5% CO_2_ and were shaken occasionally. Following the JGF treatment, 50 µl SARS-CoV-2 (2*10^3^ PFU/well) samples were added and adsorbed for 1 h at 37°C. After the absorption period, the medium was removed and 4 mL of 1.55% (v/v) methylcellulose in DMEM (2% FBS added) with JGF was added for 3 days at a temperature of 37°C in 5% CO_2_. Cells were then fixed with 10% formaldehyde for 1 h at room temperature, and 0.5% (w/v) crystal violet was added into the fixed cells for at least 30 min at room temperature. SARS-CoV-2 virus (nCoV-19/Taiwan/4/2020) was obtained from Taiwan Centers of Disease Control (CDC). All experiments involving live SARS-CoV-2 were performed in CDC-approved BSL-3 and BSL-4 facilities at the Institute of Preventive Medicine in the National Defense Medical Center in accordance with requirements of the institutional biosafety committee.

### Cell Viability Assay

Cells (5 × 10^4^ cells/well) were seeded into 12-well culture plate dishes and incubated overnight. Cells were then treated with JGF (0–800 μg/ml) for 48 and 72 h. After incubation, each well was rinsed with PBS and cells that attached to the bottom of the well were fixed and stained with 1% crystal violet solution or MTT solution as described previously (Lin et al., 2017).

### LDH Assay to Detect Cytotoxicity for Jing Guan Fang in BHK-21 and Calu-3 Cells

The cytotoxicity of JGF on BHK-21 and Calu-3 cells was determined using Cytotoxicity Detection Kit^PLUS^ (LDH; Merck). BHK-21 and Calu-3 cells were seeded in 96-well plates (1×10^4^ cells/well). After incubation overnight at 37°C, the cells were replaced into growth medium containing various concentrations of JGF (20, 40, and 80 μg/ml), and incubated at 37°C for 24 h. The untreated cells were as the low control so they spontaneously release LDH in normal condition. Cells that were treated with lysis buffer (5 μl) for 15 min were the high control and were used to determine the maximum release of LDH in the cells. To determine the LDH activity, 100 μl of Reaction mixture (freshly prepared by mixing Catalyst and Dye solution) was added to each well and incubated for 15 min at room temperature. Multimode microplate readers (TECAN SPARK) were used to measure the absorbance of the samples at a wavelength of 490 nm. To determine the percentage of cytotoxicity, the average absorbance values for three samples and controls were calculated. These values were substituted into the equation:

$$Cytotoxicity\;\left(\%\right)=\frac{exp.\;value\;-\;low\;control}{high\;control\;-\;low\;control}\times 100$$

### Sample Preparation for Western Blotting Analysis

Cells were rinsed with cold PBS containing 1% Na_3_VO_4_ and harvested by scraping the cells into proteinase inhibitors and a phosphatase inhibitor cocktail (Sigma Chemical Co.) containing lysis buffer (10 mM HEPES (pH 7.9), 10 mM KCl, 0.1 mM EDTA, 0.1 mM EGTA). Whole cell lysates were centrifuged for 13,000 xg for 10 min at 4°C. The supernatant was collected as the cell extracts. The concentration of the protein from cell lysate was determined by using a Bradford assay (Bio-Rad, Hercules, CA). The cell lysate samples (30 μg) were then subjected to western blot analysis. The expression of β-actin was used as an internal control. The procedure for the Western blotting assay used the methods and procedures that were described previously (Lin et al., 2017). Antibodies against ACE2, TMPRSS2 and actin were purchased from GeneTex (Hsinchu, Taiwan).

### RNA Extraction and Quantitative Polymerase Chain Reaction

Total RNA was isolated from cells using TRIzol reagent (Invitrogen, Carlsbad, CA, United States). The cDNA synthesis used a HiScript II 1st Strand cDNA Synthesis Kit (Vazyme, JS, China). The q-PCR used a Fast SYBR Green Master Mix (Thermo Fisher Scientific) in triplicate and an Applied Biosystems Model 7000 instrument (Thermo Fisher Scientific). The data was quantitated using 2^−ΔCt^ (ΔCt = CtTarget gene-CtGAPDH; Ct: cycle number when the fluorescent value of the sample is equal to the threshold value). Primer sequences for in this study include hTMPRSS2-F: 5′-CCT​CTA​ACT​GGT​GTG​ATG​GCG​T; hTMPRSS2-R: 5′-TGC​CAG​GAC​TTC​CTC​TGA​GAT​G-3'. hGAPDH-F: 5′-TGG​TAT​CGT​GGA​AGG​ACT​CA-3'; and hGAPDH-R: 5′-AGT​GGG​TGT​CGC​TGT​TGA​AG-3'.

### Animal Model

Six-to eight-month-old male C57BL/6 mice were purchased from the National Laboratory Animal Center (Taipei, Taiwan). To determine the effect of JGF on the expression of ACE2 and TMPRSS2 in various organs of mice, the mice were randomly sorted into experimental groups (n = 3). For Experiment 1, mice were fed with JGF (8 mg/mouse/day) twice. For Experiment 2, JGF was filled in a closed space using a low-temperature steam method (30–32°) and inhaled by mice. After exposure to JGF for 30 min, mice were moved to a normal environment for 10 minutes and then underwent another 30 min of exposure. Mice were sacrificed after receiving JGF for 3 h. All procedures were approved by and performed in accordance with the guidelines and regulations of the Institutional Animal Care and Use Committee (IACUC) of National Yang Ming Chiao Tung University (IACUC Approval NO: 1100511).

### Statistical Analysis

Statistical differences between the experimental groups were determined using a t-test in GraphPad Prism8. A vale of *p* < 0.05 indicates a statistically significant result. The experiments were conducted three times or as indicated, and all data is expressed as mean ± SD.

## Results

### Clinical Observation of Subjects Who Are Treated Using Jing Guan Fang

JGF was initially created in January 2020 for frontline medical staff and travelers who had returned from mainland China, Hong Kong, Macau and Singapore. This formula was then made available to the public in five public hospitals as an additional preventative measure. The clinical observation, involved 396 subjects who participated in the questionnaire. The average age of the participants was 45.9 years old, with a standard deviation of 14.1 years. The ratio of males to females is 35.1% (male) and 64.9% (female). Table 1 summarizes the demographic and clinical characteristics for the 396 subjects who participated in the questionnaire. Sore throat was the most reported Covid-19 like symptom, with 34 subjects displaying the symptom. Cough and headache are the second and third most commonly reported Covid-19 like symptoms and a loss of taste and smell (dysgeusia) is the least commonly reported Covid-19 like symptoms. NonCovid-19 like symptoms that were reported include fatigue (n = 128), and tension (n = 102). Seven days after taking JGF, 91.2% of subjects who experienced a sore throat reported that the symptoms had improved. Improvements in non-Covid-19 like symptoms, such as fatigue (81.3%) and tension (68.6%), were also reported.

**TABLE 1:** Demographic and clinical characteristics of the subjects with Covid-19 like symptoms.

Basic data	All (n = 396)	
Age, years (SD)	45.9 (14.1)	
Male %	35.1	
Female %	64.9	
Covid-19 like symptoms	Subjects displaying symptoms, persons (%)	Rate of improvement, persons/all persons (%)
Sore throat	34 (8.6)	31/34 (91.2)
Headache	25 (6.3)	19/25 (76.0)
Cough	29 (7.3)	23/29 (79.3)
Fever	13 (3.3)	11/13 (84.6)
Rhinorrhea	23 (5.8)	18/23 (78.2)
Diarrhea	24 (6.1)	18/24 (75.0)
New loss of taste or Smell	3 (0.8)	1/3 (33.3)
Associated main symptoms	Subjects displaying symptoms, persons (%)	Improving rate, persons/all persons (%)
Fatigue	128 (32.3)	104/128 (81.3)
Tension and pressure	102 (25.8)	70/102 (68.6)

### Jing Guan Fang Prevents Membrane Fusion and the Formation of Syncytium

**FIGURE 1:**
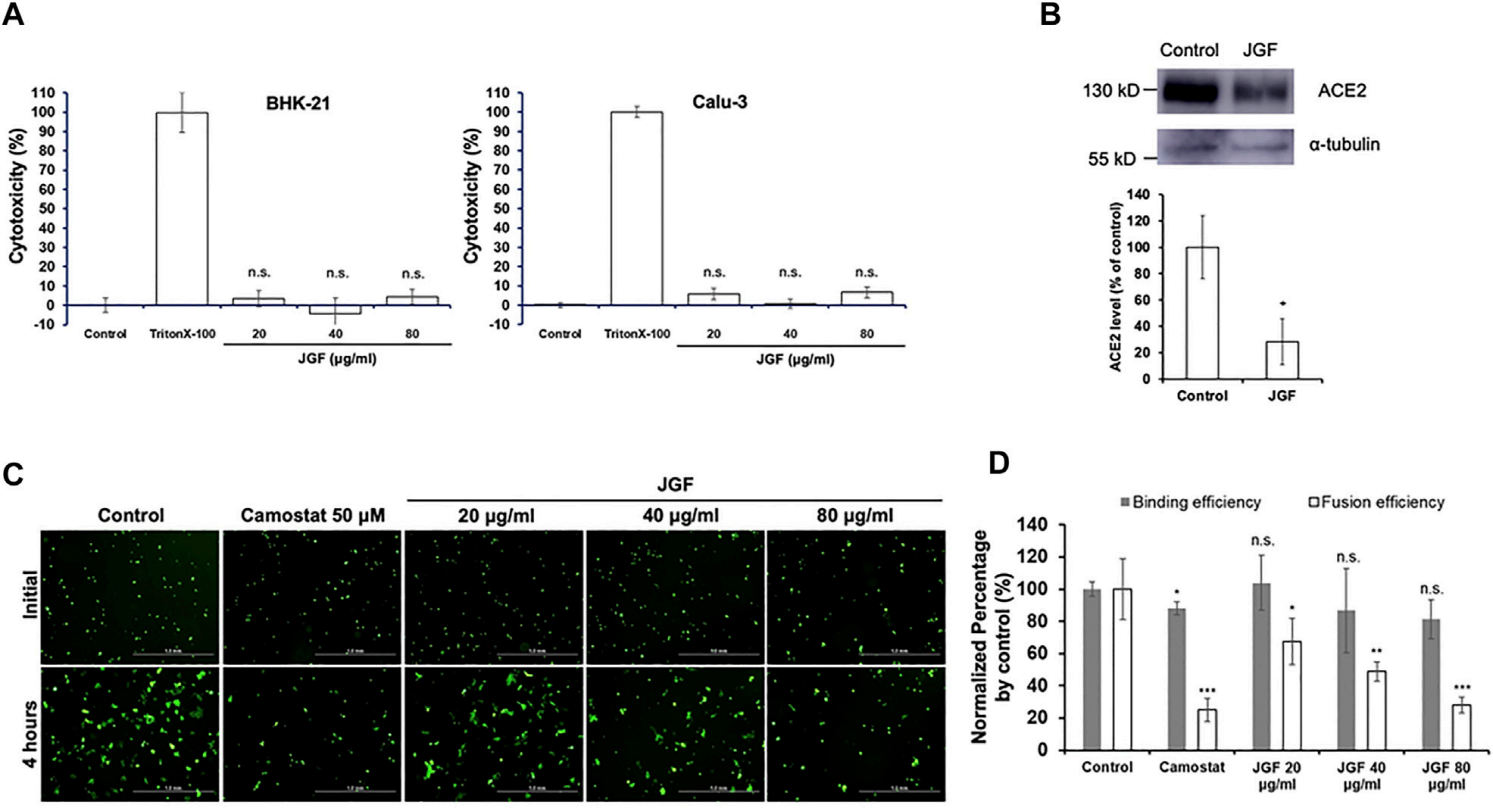
The inhibitory activity of JGF on the interactions between SARS-CoV-2 spike (S) protein and ACE2 receptor. **(A)** Validation of the cytotoxicity of JGF. BHK-21 and Calu-3 cells were treated with indicated amounts of JGF and cell viability was examined by LDH assay. The control group represented the non-treatment group, serving as a negative control. The treatment with Triton-100 was used as a positive control. The data were presented as the mean ± SD; error bars indicated SD. n. s indicated non-significant, compared to the control group. **(B)** The expression of ACE2 in Calu-3 cells were treated with 40 μg/ml of JGF was analyzed by Western blot assay (upper panel). The level of ACE2 expression was quantified after normalization with α-tubulin. The quantification analysis from three independent experiments was shown in bottom panel. * represents *p* < 0.05. **(C)** The cell-cell fusion of SARS-CoV-2 S protein-expressing BHK cells and ACE2-expressing Calu-3 cells was visualized in the presence of indicated amounts of JGF. **(D)** The formation of syncytium was quantified for various concentrations of JGF. Compared with the control, syncytium formation was significantly inhibited after 20–80 μg/ml JGF treatments. * represents *p* < 0.05.

S protein present in a cellular membrane can trigger the formation of receptor-dependent syncytia (Buchrieser et al., 2020; Cheng et al., 2020; Xia et al., 2020)so this study developed a fluorescence-based cell-cell fusion assay for which BHK cells that express both SARS-CoV-2 S protein and enhanced green fluorescent protein (GFP) act as the effectors and Calu-3 cells that express endogenous hACE2 act as targeting cells. For this assay, the binding of BHK-21 cells with Calu-3 cells indicates that SARS-CoV-2 S protein binds with the ACE2 receptor and the formation of syncytium is the results of membrane fusion. To confirm the cytotoxicity of JGF to BHK-21 and Calu-3 cells, cells were treated with various concentrations from 20 to 80 μg/ml of JGF. The treatment of Triton-100 was used as a positive control. Compared to the treatment of the control group represented the non-treatment group that served as a negative control, LDH assay depicted that JGF caused no significant amount of cell death, suggesting JGF is not cytotoxic to either BHK cells or Calu-3 cells (Figure 1A). The effect of JGF on the expression of the ACE-2 in Calu-3 cells was determined by Western blot assay. The results depicted the level of the ACE-2 was significantly decreased approximately 70% in the presence of 40 μg/ml of JGF treatment (Figure 1B). Given that the occurrence of cell-cell fusion that can be visualized as syncytium formation in the cell-cell fusion experiment, the results of confocal microscopy imaging revealed that, only in the presence of the SARS-CoV-2 S protein, DiI-labeled cellular compartments of Calu-3 cells were co-localized with GFP signals within large syncytium, suggesting that fusion of GFP-expressing BHK-21 cells with DiI-labeled Calu-3 cells (Supplementary Figure S2). In the cell-cell fusion assay, the fluorescence microscopy imaging revealed that the treatment of JGF can only inhibit the formation of syncytium but not the binding of BHK-21 cells with Calu-3 cells (Figures 1C,D). The treatment of the camostat, a pharmacological inhibitor of TMPRSS2 protease, resulting in the inhibition of syncytium formation was used as a positive control group. Moreover, the inhibition was in a dose-dependent manner, suggesting that JGF can specifically interrupt SARS-CoV-2 S-mediated membrane fusion (Figures 1C,D). These results suggest that JGF may potentially be used as an anti-SARS-CoV-2 agent because it suppresses membrane fusion, which is a key element of viral infection.

### The Cytotoxic Effect of Jing Guan Fang on Human Fibroblast WI-38 and MRC-5 Cells

**FIGURE 2:**
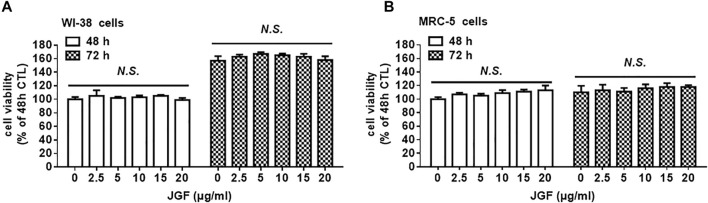
The effect of JGF on the cell viability of lung fibroblast WI-38 and MR/C-5 cells. WI-38 **(A)** and MRC-5 **(B)** cells were treated with various low concentrations of JGF (0–20 μg/ml) for 48 and 72 h. Each group of JGF-treated samples was normalized against an untreated control. Cell viability was determined using a crystal violet assay. Data were representative of three separated experiments and were presented as the mean ± SD; error bars indicated SDs.

To determine the potential mechanisms by which JGF prevents SARS-CoV-2 infection, a series of *in vitro* experiments involved human lung cells. Two human lung WI-38 and MRC-5 cells were used to determine the cytotoxic effect of JGF. A crystal violet assay was used to determine the concentration at which JGF inhibits activity in human lung cells. As shown in Figures 2A,B, we found that JGF did not exhibit the cytotoxicity to either WI-38 or MRC-5 cells.

### Jing Guan Fang Induced Lysosome-dependent Degradation of Angiotensin-Converting Enzyme-2

**FIGURE 3:**
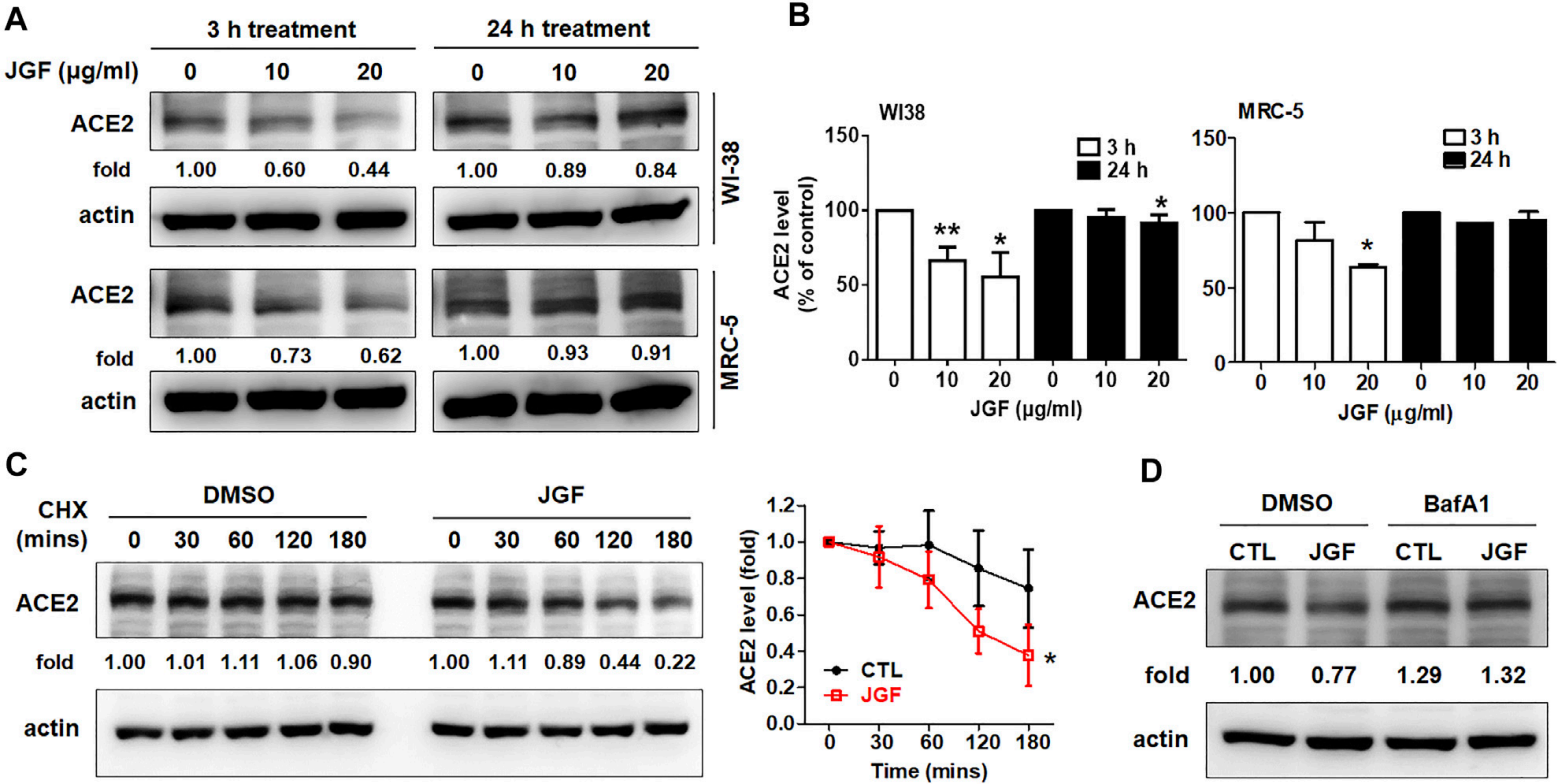
JGF induces lysosome dependent degradation of ACE2. **(A)** WI-38 and MRC-5 cells were treated with three dosages of JGF (0–20 μg/ml) for 3 and 24 h. Western blotting was subsequently performed with whole cell lysates to detect expression of ACE2. Actin was used as the internal control. **(B)** Quantification of the intensities of the bands of ACE2 was representative of three separate determinations using ImageJ (National Institute of Mental Health, Bethesda, MD, United States). The data were presented as the mean ± SD; error bars indicated SD. Significant differences were shown (*p < 0.05 compared to the control group). **(C)** Time course for ACE2 degradation after the addition of cycloheximide (CHX; 100 μg/ml) in the presence or absence of JGF (10 μg/ml) for 0–180 min in WI-38 cells as analyzed by Western blotting. Right panel: The levels of ACE2 in the three-independent experiments were quantified by ImageJ and the results were presented as the mean ± SD; error bars indicate SDs. Significant differences were shown (*p < 0.05 compared to the control DMSO group). **(D)** WI-38 cells were pretreated with DMSO (vehicle control) or BafA1 (10 μM) for 30 min, followed by incubation with JGF (10 μg/ml) for 2 h.

We found that JGF effectively interrupted the interaction between the spike protein with the ACE2 receptor and suppressed membrane fusion, which is an essential element of viral infection (Figure 1). We further examined mechanisms by which JGF inhibited infection of normal human lung cells with SARS-CoV-2. Increasing evidence shows that SARS-CoV-2 infection is prevented by targeting ACE2 in lung cells to block the SARS-CoV-2 spike receptor from binding with ACE2 (Hoffmann et al., 2020). Therefore, we investigated whether JGF affected the protein levels of ACE2 in WI-38 and MRC5 cells. As shown in Figures 3A,B, we found that brief treatment with JGF dramatically reduced the expressions of ACE2 by about 40–50%, but the ACE2 level increased during JGF treatment for a longer period. These results suggested that JGF temporarily downregulates ACE2 level to reduce the infection with SARS-CoV-2. To further explore the mechanism by which JGF downregulated ACE2, we examined the Adam17 activity because ACE2 can be cleaved into extracellular soluble ACE2 and intracellular ACE2 by Adam17 (Li et al., 2021). However, JGF did not increase the activity of Adam17 or the levels of extracellular soluble ACE2 (Supplementary Figure S3).

These results showed that JGF significantly suppressed the total protein levels of ACE2 within a short period of time. Therefore, we further examined the mechanism by which JGF downregulated ACE2 levels. Previously, ACE2 is regulated by Ang-II type 1 receptor and E3 ligase NEDD4 to respectively induce lysosome and proteasome dependent degradation of ACE2 (Deshotels et al., 2014; Ogunlade et al., 2019). We thus examined whether JGF-induced ACE2 degradation is dependent on proteasome or lysosomal systems. Initially, we analysed the half-life of ACE2 in WI-38 cells following treatment with cycloheximide (CHX). We found that when WI-38 cells were co-treated with JGF and CHX, the level of ACE2 was dramatically downregulated in a time-dependent manner (Figure 3C), suggesting that JGF could induce degradation of ACE2. Next, using the lysosome inhibitor, BafA1, we found that BafA1 recovered the ACE2 level in WI38 cells after JGF treatment (Figure 3D and Supplementary Figure S4). However, MG132, which is a proteasome inhibitor, failed to rescue JGF-induced JGF degradation (Supplementary Figure S5). These results indicated that JGF could be used to prevent SARS-CoV-2 infection by inducing ACE2 degradation.

### Jing Guan Fang Downregulates Transmembrane Serine Protease 2 Levels

**FIGURE 4:**
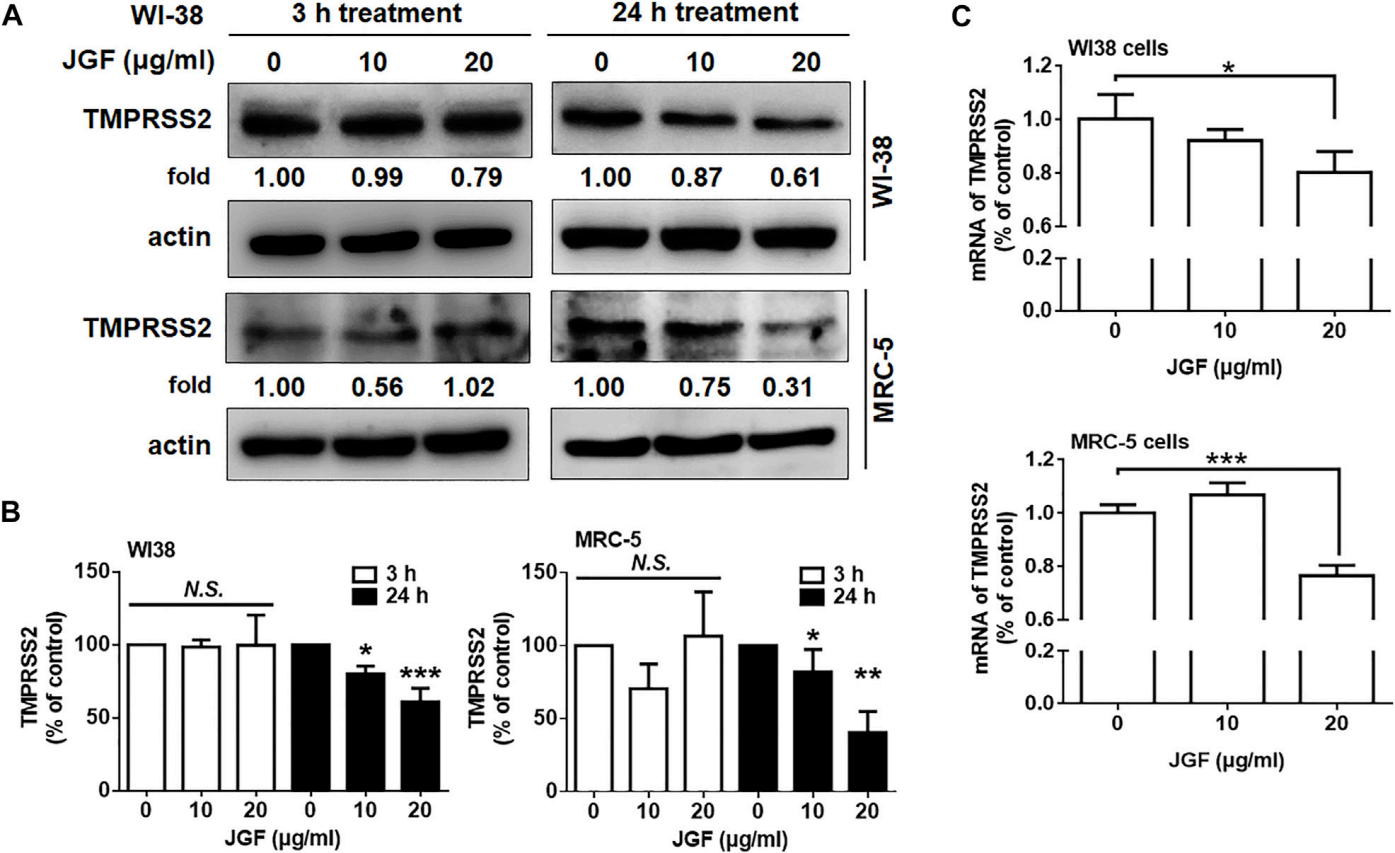
JGF reduces TMPRSS2 levels in WI-38 cells. **(A)** WI-38 cells were treated with JGF (0–20 μg/ml) for 3 and 24 h. Western blotting was subsequently performed with whole cell lysates to detect the expression of TMPRSS2. Actin was used as the internal control. **(B)** Quantification of the intensities of the bands of TMPRSS2 was representative of three separated determinations by ImageJ. **(C)** WI-38 cells were treated with JGF (0, 10 and 20 μg/ml) for 24 h. The mRNA levels of TMPRSS2 were determined by qRT-PCR. The data was presented as the mean ± SD; error bars indicated SD. Significant differences were shown (*p < 0.05 compared to the control group).

It is worthy to note that TMPRSS2 is another important membrane protein for SARS-CoV-2 infection (Dong et al., 2020; Hoffmann et al., 2020). We therefore examined whether JGF affected the expression of TMPRSS2 in WI-38 cells. As shown in Figures 4A,B, we found that JGF did not affect the expression of TMPRSS2 within a short period of time. Interestingly, long-term treatment with JGF effectively downregulated the expression of TMPRSS2 by 40–70% (Figure 4B). In parallel, JGF significantly reduced mRNA of TMPRSS2 for the 24 h treatment (Figure 4C). These results suggested that JGF may regulate the signaling transductions which controlled the synthesis of TMPRSS2.

### Jing Guan Fang Downregulates the Expressions of Angiotensin-Converting Enzyme-2 and Transmembrane Serine Protease 2 in Lung Tissue of Mice

**FIGURE 5:**
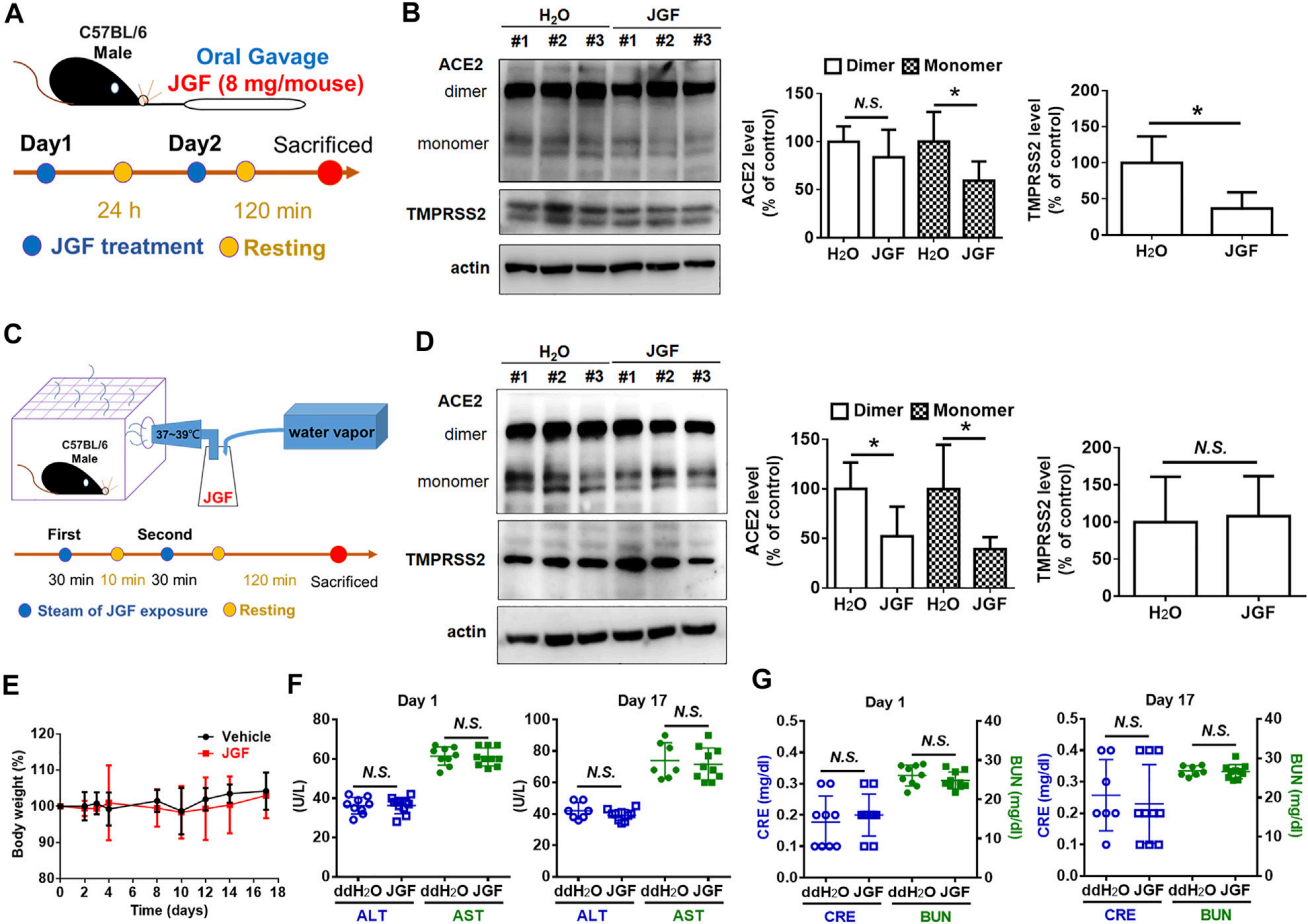
JGF reduces ACE2 and TMPRSS2 on lung tissues of mouse model. **(A)** Scheme for mouse receiving JGF via oral gavage. **(B)** ACE2 and TMPRSS2 levels in lung tissues of mice receiving JGF via oral gavage. **(C)** Scheme for mouse receiving JGF via steam spray method. **(D)** ACE2 and TMPRSS2 levels in lung tissue of mice receiving JGF via steam spray. **(E)** The body weight changes (%) for mice receiving JGF via oral gavage every other day. **(F,G)** The AST and ALT (hepatic function; **F**) as well as BUN and Creatinine (renal function; **G**) of mice sera were analyzed on Day 1 and Day 17.

It is well known that ACE2 and TMPRSS2 are expressed in human organs (Hamming et al., 2004; Hikmet et al., 2020). Specifically, ACE2 is abundantly present in the epithelia of the lung and small intestine (Hamming et al., 2004). The mRNA expressions for both ACE2 and TMPRSS2 were detected in the heart, digestive tract, kidney, and brain (Dong et al., 2020). We therefore examined the levels of ACE2 and TMPRSS2 in lung, brain, colon, and kidney tissues of mice. As shown in Supplementary Figure S6, we found that there were many isoforms of ACE2 and TMPRSS2 in these tissues. To determine whether JGF affected the levels of ACE2 and TMPRSS2 in lung tissue of mice, we conducted a series of *in vivo* experiments. Initially, we investigated the effect of orally ingested JGF on mice *in vivo*. Continuous feeding with JGF for 2 days effectively reduced protein levels of ACE2 and TMPRSS2 in the lungs of mice (Figures 5A,B). Specifically, we found that in the mice receiving the administration of JGF, the monomer of ACE levels was significantly reduced by 45%; however, the dimer form of ACE2 was not reduced in lung tissues. Surprisingly, JGF dramatically reduced protein levels of TMPRSS2 by 50% (Figure 5B). Moreover, in the view of traditional Chinese medicine, drugs could be absorbed through the nasal cavity by steam method, which can make the herbal medicine quickly enter the nasal cavity and lungs to address cold symptoms. We thus used the steam method to induce the mice to inhale JGF (Figure 5C). As shown in Figure 5D, we found that ACE2 was dramatically reduced by more than 50% after the mice inhale JGF; however, TMPRSS2 levels were unchanged after the short-term exposure to JGF. Alternatively, we found JGF did not affect body weight of mice (Figure 5E). Furthermore, to examine the degree of liver and kidney injury in mice that were fed with JGF, the AST/ALT and BUN/creatinine levels were analyzed in blood samples at the end of JGF treatment. Our results indicated neither evidence of JGF affecting liver functions nor kidney toxicity in the mouse model (Figures 5F,G). These results suggest that JGF may reduce infection with SARS-CoV-2 without side effect.

### Jing Guan Fang inhibits the formation of plaque formation for The Severe Acute Respiratory Syndrome CoronaVirus 2 on Vero E6 cells.

**FIGURE 6:**
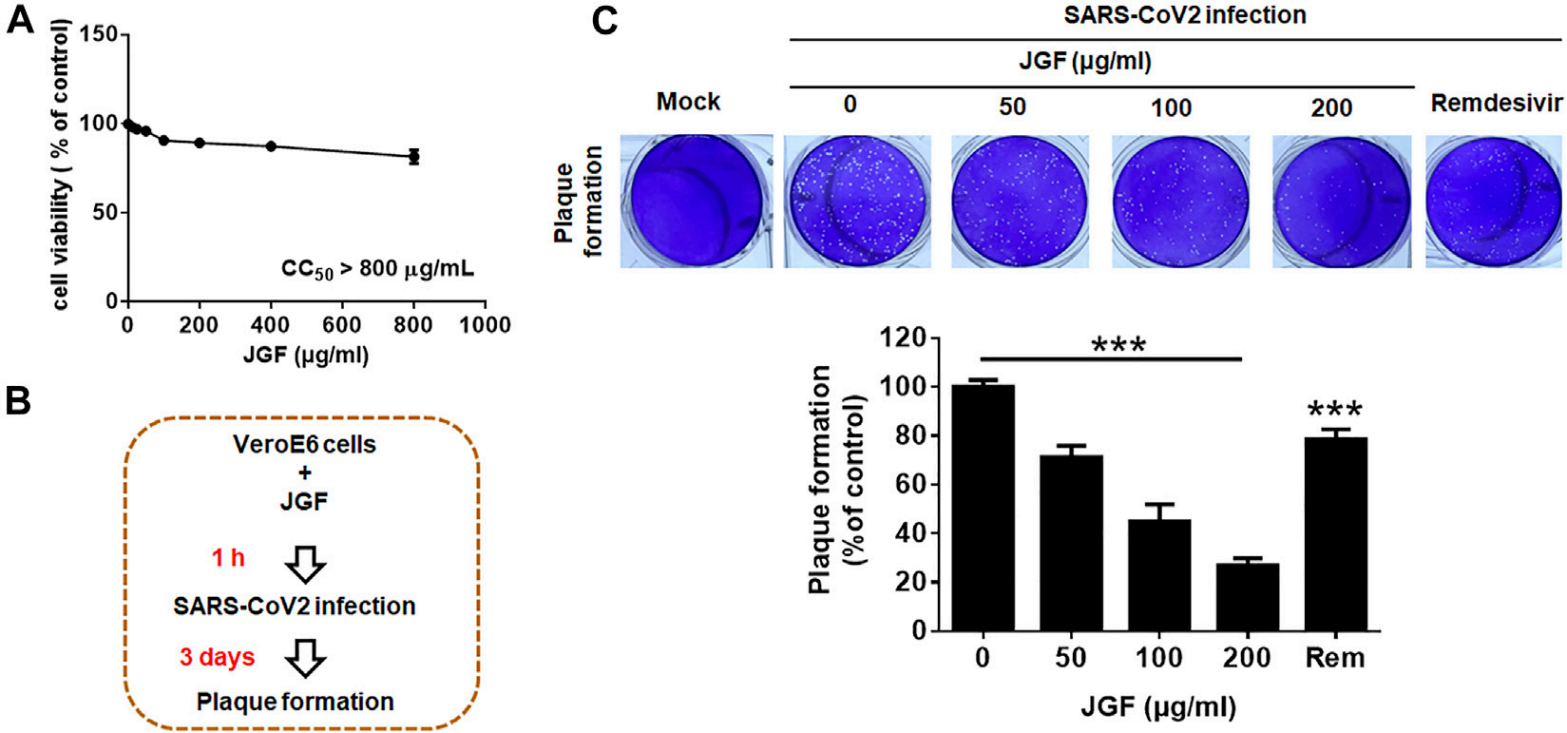
JGF inhibits plaque formation for SARS-CoV-2 in Vero E6 cells. **(A)** Vero E6 cells were treated with various concentrations of JGF (0–800 μg/ml) for 72 h. Each group of JGF-treated samples was normalized against an untreated control. Cell viability was determined using the MTT assay. **(B)** Vero E6 cells were pre-treated with JGF (0–200 μg/ml) for 3 h and then infected with SARS-CoV-2 for 3 days **(C)** The plaque was stained with crystal violet. Data were representative of three separate experiments and were presented as the mean ± SD; error bars indicated SDs.

The above showed that JGF can prevent viruses from infecting cells by reducing ACE2 and TMPRSS2. Therefore, we further investigated whether JGF could inhibit the viral infection and proliferation. The Vero E6 cells were chosen for investigating the SARS-CoV-2 infection. Initially, we examined the effects of JGF on cell viability of Vero E6 cells. As shown in Figure 6A, we found JGF did not exhibit a cytotoxic effect on Vero E6 cells even at high concentration of JGF. A cytotoxic concentration of 50 (CC_50_) of JGF was more than 800 μg/ml. Next, virus plaque formation assay was conducted to examine the efficacy of JGF in preventing SARS-CoV-2 infection. Vero E6 cells were pretreated with JGF before SARS-CoV-2 infection (Figure 6B). We found that JGF dramatically inhibited plaque formation in a concentration-dependent manner compared to remdersivir (2 μM; Figure 6C). Specifically, JGF at 200 μg/ml dramatically reduced plaque formation of SARS-CoV-2 by 70%. Taken together, these results suggest that JGF may reduce SARS-CoV-2 infection and proliferation.

## Discussion

The current COVID-19 epidemic is a worldwide problem so if the viral infection can be effectively prevented, the disease rate will be reduced. This study mainly provides a scientific evidence-based view of the mechanism by which JGF reduces the infection and proliferation of SARS-CoV-2. Moreover, we demonstrated that JGF did not inhibit cell viability for normal lung cells *in vitro* and did not induce a toxic effect on liver and kidney of mice *in vivo*. The clinical observations demonstrated that JGF treatment improved the COVID-19 like symptoms. This study combines basic research and clinical observation to explore the effects of and the mechanism by which JGF prevents SARS-CoV-2 infection. Future clinical trials of the efficacy of JGF in attenuating viral infection are further evaluated.

According to the epidemiological theory of Traditional Chinese Medicine and clinical experience of SARS (Hsu et al., 2008; Lin, 2020; Hsu et al., 2006a; Hsu et al., 2006b), this study uses five common herbal medicines to formulate JGF using a specific ratio. In this study, we focused on the effects and the mechanism of the JGF formula but not individual herbal medicines because we believed that using all five herbs together may provide multifactorial effects that a single compound or herb may not be able to provide. Increasing evidence shows that these five herbal medicines exhibit anti-viral effects. Phillyrin (KD-1), which is isolated from *Forsythia suspense*, inhibits SARS-CoV-2 replication and inflammatory factors that are caused by viral infection (Ma et al., 2020). Baicalein, which is purified from *Scutellaria baicalensis* Georgi, inhibits SARS-CoV-2 replication (Huang et al., 2020). The extract from *Bupleurum chinense* DC exhibits anti-SARS-CoV-2 effects (Wu et al., 2020). Extracts from *Magnolia officinalis* and *Agastache rugose* have an anti-inflammatory effect (Seo et al., 2019; Ren et al., 2021). However, no studies show whether these herbs prevent SARS-CoV-2 infection. To examine the effect of JGF on SARS-CoV-2 infection, we initially developed a fluorescence-based cell-cell fusion assay in which BHK cells that express SARS-CoV-2-spike protein and EFGP act as the effectors, and Calu-3 cells expressing endogenous hACE2 act as targeting cells because SARS-CoV-2 cell infection depends on ACE2 and TMPRSS2 (Hoffmann et al., 2020). The results show that pretreatment with JGF of Calu-3 cells with JGF dramatically inhibits the formation of syncytium. In addition, pretreatment with JGF effectively inhibited the formation of virus plaque for SARS-CoV-2 on Vero E6 cells. Therefore, we further examined whether JGF affected the interaction between SARS-CoV-2-spike protein and hACE2. The results for the ACE2/Spike protein interaction assay kit showed that JGF slightly interrupted the affinity of the SARS-CoV-2 spike for human ACE2 receptor in a dose-dependent manner. JGF inhibited approximately 20% of the interaction between spike and ACE2 at a concentration of 200 μg/ml (data not shown). These results suggest that JGF has a pivotal role in preventing the infection with SARS-CoV-2. Targeting on the interaction between SARS-CoV-2-spike protein and hACE2 may not reflect the main effect of JGF.

To examine the mechanism by which JGF prevented infection with SARS-CoV-2, we focused on the effects of the JGF-regulated expressions of ACE2 and TMPRSS2. Using the normal lung WI-38 2RA and MRC-5 cells, we found that JGF effectively reduced ACE2 and TMPRSS2 levels in the short and long-time treatment, respectively. Previous studies showed that ACE2 plays a pivotal role in SARS-CoV-2 cell entry (Hoffmann et al., 2020; Jia et al., 2021), so we focused on how JGF reduced ACE2 levels in normal lung cells. Soluble ACE2 (sACE2) from ACE2 cleaved by ADAM17 is used as a decoy receptor to trap spikes of virus to prevent cellular engagement for COVID-19 therapy (Monteil et al., 2020; Jia et al., 2009). However, we found that JGF did not significantly induce expression of sACE2 and activate ADAM17 Supplementary Figure S3. In contrast, JGF temporarily caused ACE2 to follow a lysosome-dependent degradation pathway, but long-term treatment allows the ACE2 protein would recover to its normal level. ACE2 is a multifunctional protein that controls physiological and pathological regulation (Baratchian et al., 2021; Li et al., 2020; Zipeto et al., 2020), so these results indicated that JGF does not cause long-term reduction in ACE2, which may prevent side effects that were caused by the long-term inhibition of ACE2 expression. However, TMPRSS2-mediated ACE2 cytoplasmic tail cleavage is correlated to enhanced viral uptake (Heurich et al., 2014). Interestingly, we found that long-term treatment with JGF could regulate mRNA level of TMPRSS2, leading to reduction of TMPRSS2 protein levels. The effect of JGF on the androgen receptor (AR)-mediated TMPRSS2 (Li et al., 2021) needs to be investigated in the future. Together, according to the concept of a cocktail therapy, we believed that JGF has at least two effects: the first is to temporarily induce ACE2 degradation, this study shows that JGF temporarily induces ACE2 degradation and reduces transcription of TMPRSS2. Currently, no studies show that traditional Chinese medicine reduces the expression of ACE2 and TMPRSS2 in host cells. In the future, it is needed to further identify the potential molecules of these herbs that regulate the expression of ACE2 and TMPRSS2 in host cells.

## Conclusion

This study used TCM theory to formulate an herbal medicine formula, JGF, as an adjuvant preventive strategy against SARS-CoV-2 infection in addition to the use of vaccines. We provided the evidence and demonstrated that JGF effectively blocked the formation of syncytium and inhibited the formation of SARS-CoV-2 plaque. In addition, we identified the potential mechanisms of JGF-reduced SARS-CoV-2 invasion by induction of lysosome-dependent ACE2 degradation and the inhibition of TMPRSS2 expression. These results suggest that JGF may be a promising formula to alleviate SARS-CoV-2 infection. In Taiwan, a research group has recently developed a traditional Chinese medicine formula called NRICM101 which is mainly used for hospitalized patients with COVID-19. This medicine reduces the number of viruses and suppresses immune storms (Tsai et al., 2021). In this study, we show that JGF reduces viral infections for frontline staff who have intensive contact with infected patients. Results from the study suggest that JGF can be an useful preventative measure for frontline medical staff or people who have had high-risk exposure to COVID-19 cases.

## Data Availability

The raw data supporting the conclusions of this article will be made available by the authors, without undue reservation.

## Abbreviations

SARS-CoV-2, the severe acute respiratory syndrome coronavirus 2; COVID-19, the coronavirus disease 2019; MERS-CoV, middle east respiratory syndrome coronavirus; RBD, receptor binding domain; ACE2, angiotensin-converting enzyme-2; TMPRSS2, transmembrane serine protease 2; TCM, traditional Chinese medicine; JGF, jing guan fang; HPLC, high-performance liquid chromatography; FBS, fetal bovine serum; DMEM, Dulbecco’s modified eagle medium; MTT, 3 (4,5-Dimethylthiazol-2-yl)-2,5-diphenyltetrazolium bromide.
