# Corrigendum: The traditional Chinese medicine formula Jing Guan Fang for preventing SARS-CoV-2 infection: from clinical observation to basic research

**DOI:** 10.3389/fphar.2023.1272615

**Published:** 2023-08-28

**Authors:** Yueh-Hsin Ping, Hsin Yeh, Li-Wei Chu, Zhi-Hu Lin, Yin-Chieh Hsu, Lie-Chwen Lin, Chung-Hua Hsu, Shu-Ling Fu, Tung-Yi Lin

**Affiliations:** ^1^ Department and Institute of Pharmacology, National Yang Ming Chiao Tung University, Taipei, Taiwan; ^2^ Institute of Biophotonics, National Yang Ming Chiao Tung University, Taipei, Taiwan; ^3^ Institute of Traditional Medicine, National Yang Ming Chiao Tung University, Taipei, Taiwan; ^4^ National Research Institute of Chinese Medicine, Taipei, Taiwan; ^5^ Branch of Linsen Chinese and Kunming, Taipei City Hospital, Taipei, Taiwan; ^6^ Biomedical Industry Ph.D. Program, National Yang Ming Chiao Tung University, Taipei, Taiwan

**Keywords:** Chinese medicine decoction, Jing Guan Fang, ACE2, TMPRSS2, COVID-19, prevention

In the published article, there was an error. The formula listed for JGF was incorrect.

A correction has been made to **Materials and Methods**, *Jing Guan Fang Preparation*, paragraph one. The incorrect sentence previously stated:

“The formula for JGF uses five herbs: 10 mg of *Forsythia suspensa* (30.3% of total weight), 8 mg of *Scutellaria baicalensis* (24.2% of total weight), 6 mg of *Bupleurum chinese* (18.2% of total weight), 6 mg of *Magnolia officinalis* (18.2% of total weight) and 3 mg of *Agastache rugose* (9.0% of total weight).”

The corrected sentence appears below:

“The formula for JGF uses five herbs: 10 g of *Forsythia suspensa* (Thunb.) Vahl [Oleaceae] (30.3% of total weight), 8 g of *Scutellaria baicalensis* Georgi [Labiatae] (24.2% of total weight), 6 g of *Bupleurum chinense* DC. [Umbelliferae] (18.2% of total weight), 6 g of *Magnolia officinalis* Rehder & E.H.Wilson var. *biloba* Rehder & E.H.Wilson [Magnoliaceae] (18.2% of total weight) and 3 g of *Agastache rugosa* (Fisch. & C.A.Mey.) Kuntze [Labiatae] (9.0% of total weight).”

The authors apologize for this error and state that this does not change the scientific conclusions of the article in any way. The original article has been updated.

