# Supplementary material for: The Traditional Chinese Medicine Formula Jing Guan Fang for Preventing SARS-CoV-2 Infection: From Clinical Observation to Basic Research
Source: Front Pharmacol. 2022 Mar 21;13:744439. doi: 10.3389/fphar.2022.744439 (PMC8978714; doi:10.3389/fphar.2022.744439)
Supplement: Supplementary file 1 [file Presentation1.pdf]

The Traditional Chinese Medicine Formula Jing Guan Fang for preventing SARS-CoV-2 infection: from clinical observation to basic research

Yueh-Hsin Ping, Hsin Yeh, Li-Wei Chu, Zhi-Hu Lin, Yin-Chieh Hsu,
Lie-Chwen Lin, Chung-Hua Hsu, Shu-Ling Fu, Tung-Yi Lin

Supplementary information

Supplementary method

HPLC analysis of JGF

The JGF decoction was freeze-dried with a Freeze dryer system (FD4.5-8P-D; Kingmech scientific co., LTD., Taiwan), yielding the dry powder of JGF. A 10.1 mg of dry JGF was dissolved in 2 mL 50% ethanol and vortexed for 30 s. This solution was filtrated by syringe filter (0.4 μm), then the filtrate was transferred to HPLC analysis. The HPLC fingerprint of JGF was performed on a Shimadzu HPLC system (Kyoto, Japan), consisting of a LC-20AT pump, a SIL-20A autosampler equipped with a 100-μL sample loop, a SPA-M20A photodiode array detector and a LabSolution Software chromatographic data system. Separation of JGF was performed on a reverse-phase C18 column (COSMOSIL 5C18-AR-II, 4.6 × 250 mm, 5 μm, Nacalai Tesque INC., Kyoto, Japan). Mobile phases consisted of solvents A (water with 0.1% formic acid) and B (acetonitrile). An eluting program was performed, from 10 to 60% B over 40 min, then increased to 90 % B in the next 1 minute, and finally maintained 90 % B to 50 min at flow rate 1.0 mL/min. An aliquot (10 μL) of sample was injected for analysis and the profile was recorded at UV 254 nm.

Adam17 activity assay

For Adam17 enzyme activity assay, TACE inhibitor screening assay kit (Biovision, catalog no. K366) was used as protocol with modification. Briefly, JGF (0, 5, 10 and 20 μg/ml) and GM6001 (100 μM, as positive control) were pretreated with purified Adam17 for 1 h at 37℃. After adding the substrate of Adam17, the mixture was placed in ELISA reader (Varioskan LUX, Thermofisher) kept at 37℃ and record the fluorescence (Ex/Em = 318/449 nm) every half minutes for 1 h.

Supplementary Figures

Supplementary Figure 1.

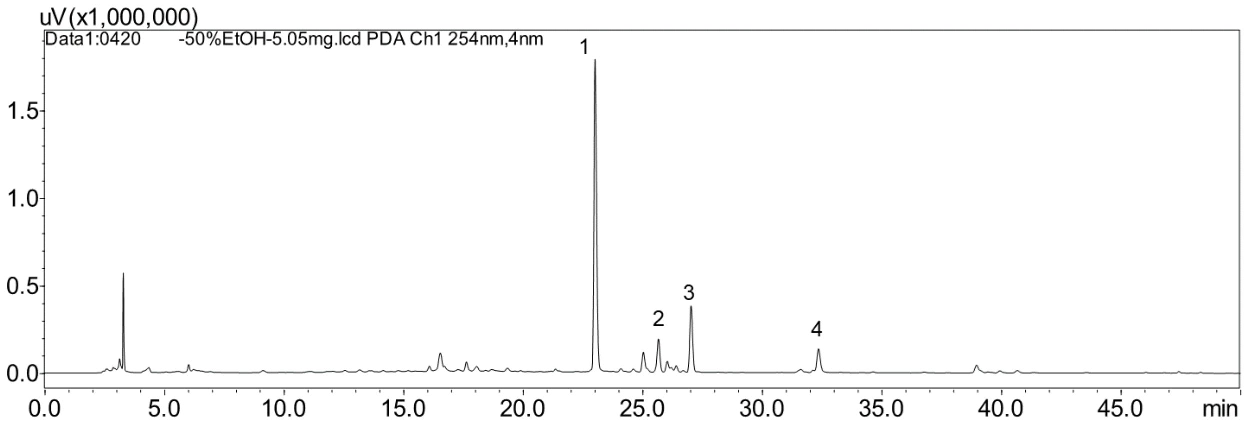

Supplementary Figure 1. The HPLC fingerprint of JGF under UV 254 nm detection. peak 1: baicalin, rt=23.0 min; peak 2: wagonin-7-*O*-glucuronide, rt=25.6 min; peak 3: wagonin-7-*O*-glucose, rt=27.0 min; peak 4: baicalein, rt=32.4 min

Supplementary Figure 2.

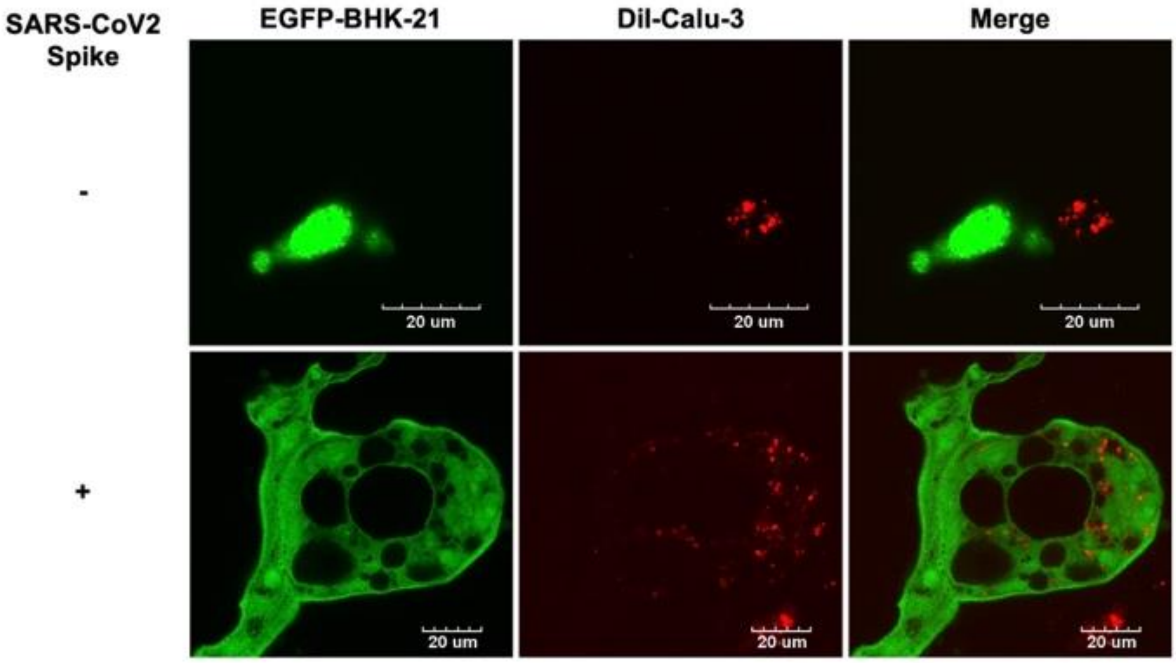

Supplementary Figure 2. The confocal images depicted EGFP expressing BHK-21 cells as green and DiI-labeled Calu-3 cells as red. In the absence of SARS-CoV-2 S protein, EGFP-expressing BHK-21 cells could attach but not fuse with Calu-3 cells. While EGFP and SARS-CoV-2 Spike co-expressing in BHK-21 cells, BHK-21 could fuse with DiI-labeled Calu-3 cells to form a large syncytium containing both EGFP and DiI signals together.

Supplementary Figure 3.

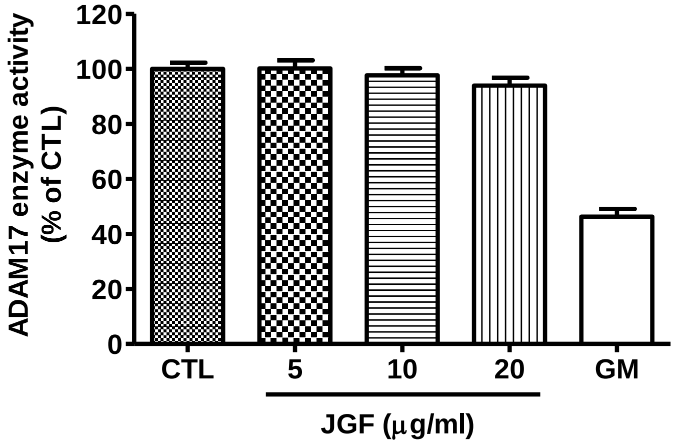

Supplementary Figure 3. JGF (0, 5, 10 and 20 μg/ml) and GM6001 (GM; 100 μM) were pretreated with purified Adam17 for 1 h at 37℃. The activity of Adam17 was measured by TACE inhibitor screening assay kit. Data were representative of three separated experiments and were presented as the mean ± SD; error bars indicated SDs. GM6001 was as positive control.

Supplementary Figure 4.

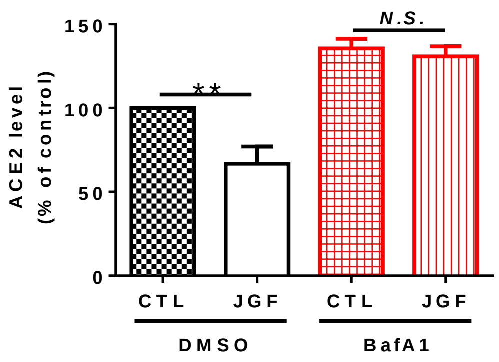

Supplementary Figure 4. Quantification of ACE2 proteins levels in Figure 3D. Quantification of the intensities of the bands of ACE2 in the Figure 3D was the representative of three separate determinations by ImageJ (National Institute of Mental Health, Bethesda, MD, USA). The data were presented as the mean ± SD; error bars indicate SD. Significant differences were shown (***P* < 0.01, compared with the control group).

Supplementary Figure 5.

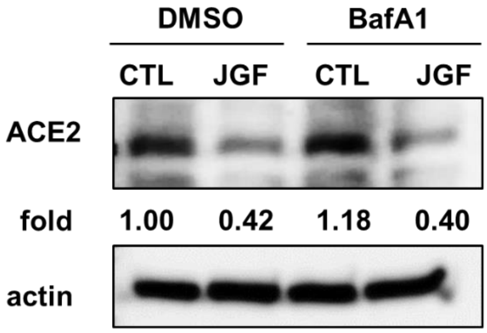

Supplementary Figure 5. WI-38 cells were pretreated with DMSO (vehicle control) or MG132 (10 μM) for 30 min, followed by incubation with JGF (10 μg/ml) for 2 h. Western blotting was subsequently performed with whole cell lysates to detect expression of ACE2. Actin was used as the internal control.

Supplementary Figure 6.

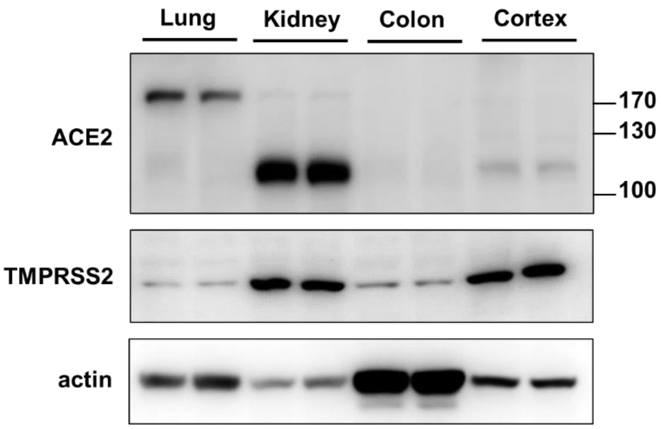

Supplementary Figure 6. The expressions of ACE2 and TMPRSS2 in various organs of C57BL/6 mouse. Two 6 weeks old mice were sacrificed and harvested various organs to evaluate the levels of ACE2 and TMPRSS2. The expressions of indicated proteins were determined by Western blot assay. Actin was used as the internal control.
